# Aphasic Dystextia as Presenting Feature of Ischemic Stroke in a Pediatric Patient

**DOI:** 10.1155/2016/3406038

**Published:** 2016-08-07

**Authors:** Arpita Lakhotia, Alok Sachdeva, Supriya Mahajan, Nancy Bass

**Affiliations:** ^1^Rainbow Babies and Children's Hospital, 11100 Euclid Avenue, Cleveland, OH 44106, USA; ^2^University Hospitals Case Medical Center, 11100 Euclid Avenue, Cleveland, OH 44106, USA

## Abstract

Aphasia is an important presenting symptom of acute stroke. With increasing reliance on electronic communication, incoherent texting or “dystextia,” which is a subset of aphasia that is reflected in text messages, can be a useful tool for symptom recognition and analysis. It can be a red flag for the family and therefore can help in early identification of an acute neurological deficit. It is also useful for providers to reliably analyze the deficit as well as establish a timeline of evolution of symptoms. There have been case reports where dystextia has been the presenting feature of stroke or complicated migraine and in one case of meningioma. We present the case of a teenage patient that in our knowledge is the youngest reported case of dystextia, whose aphasia recorded in a text message assisted with stroke localization. This also adds to the literature of dystextia which so far has only seven other cases reported.

## 1. Introduction

Acute stroke is a neurological emergency and needs quick diagnosis to ensure appropriate intervention. Aphasia is an important presenting symptom of stroke. Dystextia or incoherent text messaging particularly when deviating from the usual norms of texting for an individual is an emerging subset of aphasia [1]. There are scattered case reports of adults where dystextia is the presenting symptom of a focal neurological deficit [1–7]. There have been no cases reported with this finding in the pediatric population. With the ubiquitous use of electronic texting devices in the teenage population, aphasia can reflect in text messages as incoherent messages. Such messages can not only provide an extra clue and aid in diagnosis, but can also provide information regarding time of onset and a rough timeline of progression of symptoms.

## 2. Case Presentation

**Figure 1:**
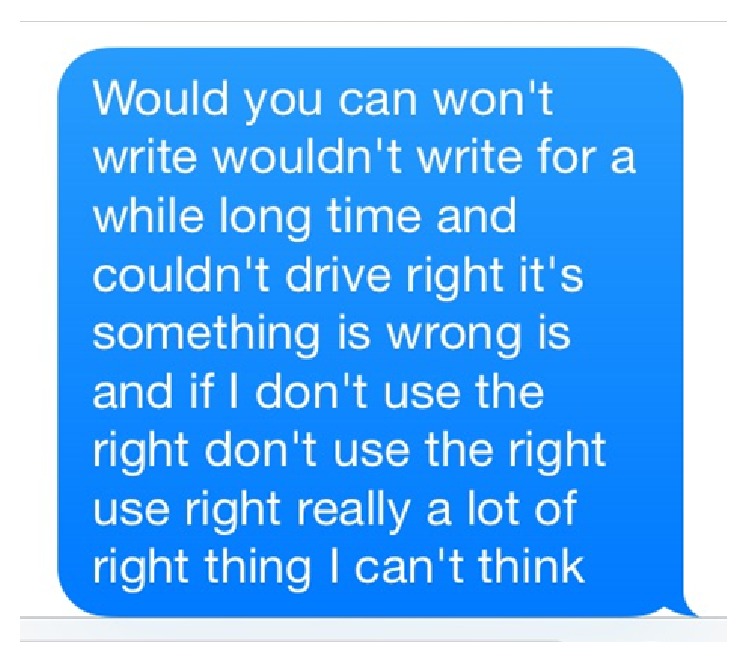
Text message sent at the time of stroke (autocorrection: on).

A 17-year-old right-handed girl who had no medical history presented to the Emergency Department (ED) after waking up from sleep with neurologic deficits. She had gone to bed in her usual state of health and awakened from sleep at 0600. She recognized right hand impairment with clumsiness when she tried to plug in the phone charger and open the door of the room. Mother opened the door to her room and tried talking to her but realized that the patient could not form coherent sentences. Therefore, her mother asked her to text her and explain what she meant. She sent her mother a text message at 0614 which read as follows: “*Would you can won't write wouldn't write for a while long time and couldn't drive right it's something is wrong is and if I don't use the right don't use the right use right really a lot of right thing I can't think.” *(Figure 1) Due to the inability to talk and text coherently, she was brought into the ED. She had stable vitals at presentation. Neurological examination revealed nonfluent aphasia with anomia and phonemic paraphasias, with impaired pinprick sensation in the right forearm. Strength was intact in all extremities at the time of examination. No acute therapeutic intervention was made due to unknown time of onset and low National Institute of Health (NIH) stroke scale score. She was admitted to the hospital for evaluation of the etiology of stroke.

**Figure 2:**
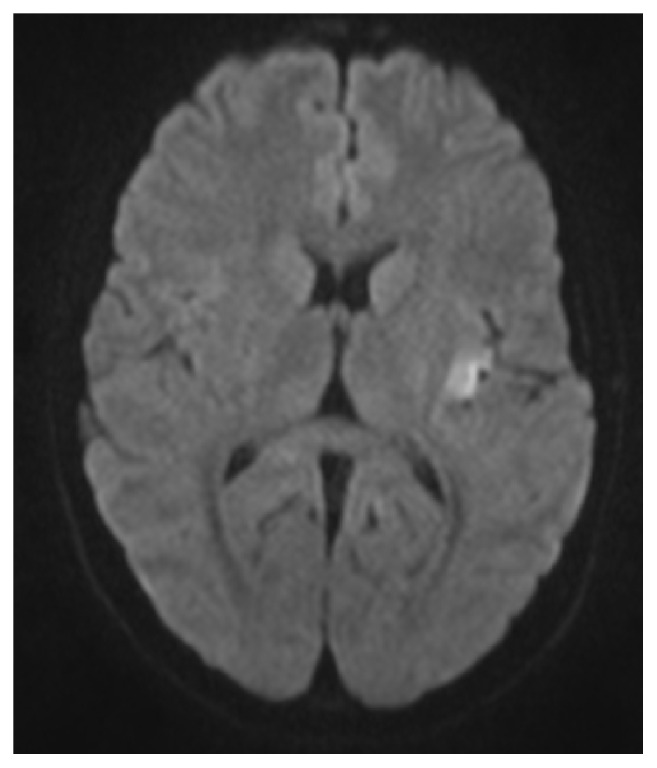
MRI brain: area of diffusion restriction in the left insula.

**Figure 3:**
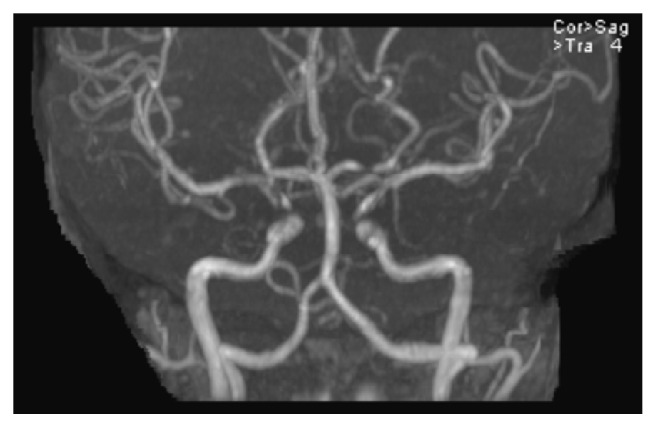
MRA: diminished flow in anterior division of left middle cerebral artery.

A Magnetic Resonance Imaging (MRI) scan of the brain showed diffusion restriction in the left insula and parietal cortex suggestive of acute/subacute ischemic stroke (Figure 2). Magnetic Resonance Angiography (MRA) revealed occlusion of distal left middle cerebral artery branch with possible stenosis in the left posterior cerebral artery (Figure 3). Medication history revealed recent use of oral contraceptive pills (OCPs) containing combination estrogen/progesterone which were started in the preceding month. Drug screen was negative. Prothrombotic workup was normal. Vascular ultrasounds showed no evidence of deep vein thrombosis. Transthoracic echocardiogram with bubble study showed a small patent foramen ovale (PFO).

OCPs were discontinued, and patient was started on low dose aspirin. Deficits rapidly resolved over the next few days. Due to the presence of PFO and no clear etiology of stroke, family chose to undergo PFO closure which was performed three months after the stroke with a septal occluder. Neurological deficits rapidly resolved after the acute presentation and on a follow-up visit four months later she only had intermittent word finding difficulty.

## 3. Discussion

“Dystextia” has been defined as inability to coherently text. Its first coinage was noted in the 2006 paper by Cawood et al. [1] where decreased speed and accuracy of sending text messages were a part of presenting symptoms of a patient. This was found to be due to infarction in the genu of the internal capsule and was a motor phenomenon of decreased dexterity. Since then it has been used in the context of both receptive and expressive aphasia in six other published reports (Table 1). The etiology in four of these was due to acute stroke [3–6]. While dystextia was not the sole manifesting feature of the acute deficit in any of the cases reported, in a few of these reports it aided in early diagnosis and helped form management decision [3, 5, 6]. In two of the cases, it aided the family in recognizing changes from the neurological baseline and alerted the emergency services [3–6]. In one case looking through the string of text messages and identifying the time point at which change occurred in texting pattern aided in establishing the exact onset of symptoms sometimes referred to as the “time of last normal” and therefore thrombolysis for acute stroke [5].

With current trends, in the teenage and young adult population, communication by text message and social media has become ubiquitous, with up to 91% sending daily mobile messages [8]. While strokes are less common in this population, it is well reported that there is often a delay in diagnosis in this subset due to lack of an index of suspicion [9]. For family members, recognizing a change from the baseline texting habits can be of neurological etiology which is important as this can bring the patient to medical attention sooner. As medical care provider, use of the mobile device as an additional tool for identification of symptoms in the appropriate patient can accelerate the time to diagnosis and initiate possible intervention if indicated. This is extremely important as not only can it aid in detection of the deficit, but also with the presence of timestamps on messages a determination of time of onset of the deficit can be made.

Limitation of using dystextia as a medical sign is the lack of rules and regulations in texting in general which has less grammatical restrictions than written or spoken language. This is why it becomes important to identify deviation from normal texting for an individual as a neurological sign and assess for other neurological deficits. There are certain nonpathological conditions as well that can lead to dystextia such as “drunk texting” or issues with the autocorrect function on mobile phone. This should also be kept in mind when using dystextia as a tool for diagnosis.

## 4. Conclusions

With emerging means of communication and increasing use of mobile messaging systems, dystextia can be a red flag for family and friends to identify a neurologic deficit and seek help. It can also help the medical provider characterize the patient's deficits as well as establish time of onset, thus aiding with treatment decisions especially in acute stroke.

## Figures and Tables

**Table 1:** Literature review of cases with dystextia.

Year, author	Age	Etiology	Text presentation	Type of dystextia	Other deficits	Imaging	Highlights
2006, Cawood et al. [1]	40 years	Stroke	Decreased speed and accuracy	Left arm weakness, loss of dexterity	L sided facial weakness and slurring	Infarction genu of R internal capsule	First reported use of the term “dystextia”
2011, Whitfield and Jayathissa [2]	20 years	Migraine	Inability to compose text message	Aphasia (expressive)	Headache, nausea/vomiting, expressive dysphasia	Normal imaging	
2013, Ravi et al. [3]	25 years	Stroke	Incoherent text message	Aphasia (receptive)	R hemiparesis, R sensory loss, fluent dysphasia	L insula stroke; poor flow in inferior division of L MCA	Aided family in recognizing stroke symptoms
2014, Burns and Randall [5]	18 years	Stroke	Incoherent texts	Aphasia (expressive + receptive)	Severe dysphasia, visual field cut	CT: hyperdensity in R lentiform nucleus and caudate	Aided in obtaining time for thrombolysis
2013, Kaskar et al. (Poster) [4]	40 years	Stroke	Disjointed text messages	Aphasia (expressive)	Slurred speech, R facial weakness		
2014, Al Hadidi et al. [6]	61 years	Stroke	Incoherent text messages	Aphasia	R hand clumsiness, inability to read TV, bilateral carotid bruits	Large L MCA stroke; severe L ICA stenosis	Aided family in recognizing symptoms
2014, Hannah et al. [7]	36 years	Meningioma	Frequent, decreased accuracy of text messages	Aphasia (expressive)	Depression, headache, altered mental status	R frontal meningioma	
